# A global database of intentionally deployed wrecks to serve as artificial reefs

**DOI:** 10.1016/j.dib.2018.12.023

**Published:** 2018-12-29

**Authors:** Iglika Ilieva, Lionel Jouvet, Lars Seidelin, Benjamin D. Best, Sofia Aldabet, Rita da Silva, Dalia A. Conde

**Affiliations:** a Biology Department, University of Southern Denmark, Campusvej 55, 5230 Odense, Denmark; b Interdisciplinary Centre on Population Dynamics, University of Southern Denmark, Campusvej 55, 5230 Odense, Denmark; c Species360 Conservation Science Alliance, 7900 International Drive, Suite 1040, Bloomington, MN 55425, USA; d EcoQuants LLC, 508 E Haley St, Santa Barbara, CA 93103, USA; e Department of Physical Geography and Ecosystem Science, Lund University, Sölvegatan 12, S-223 62 Lund, Sweden

**Keywords:** Artificial reef, Shipwreck, Sunken vessels, Wrecks as reefs, Biodiversity, Conservation

## Abstract

This paper contains data on intentionally deployed wrecks to serve as artificial reefs from 1942 to 2016. The deployment of decommissioned vessels and other available wrecks is a common practice in many coastal countries, such as the USA, Australia, Malta, and New Zealand. We obtained data of georeferenced sites of wrecks from the scientific literature, local databases, and diving web sites published in the English language. Furthermore, we included information regarding the type of structure, location, depth, country, year of deployment and estimated life span. Moreover, we provide information on whether the wreck is located inside one of the World׳s Protected Areas, key biophysical Standard Level Data from the World Ocean Database, distance to reefs from the Coral Trait Database, and distances to 597 aquariums that are members of the Species360 global network of Aquariums and Zoological institutions, in the Zoological Information Management System (ZIMS). We provide data for wrecks with monitoring surveys in the peer-review literature, although these only comprise 2% of the records (36 of 1907 wrecks). The data we provide here can be used for research and evaluation of already deployed reefs, especially if combined with additional spatial information on biodiversity and threats.

**Specifications table**

**Table:** 

Subject area	*Biology*
More specific subject area	*Fisheries, artificial reefs, corals, aquariums, wrecks, conservation*
Type of data	*Tables, graphs, figures*
How data was acquired	*From published literature and websites in the English language*
Data format	*csv file, digitalized, standardized, and analysed data*
Experimental factors	*The data were compiled, digested and standardized from diverse sources, including local artificial reef databases, scientific publications, diving web sites and the Species360׳s ZIMS. We estimated age of wrecks based on the material from 39 studies. Associated key biophysical Standard Level Data and estimates of distances to the World Protected Areas and Aquariums*
Experimental features	*We built the database in R based on currently available online data, including published literature and websites in the English language*
Data source location	*The data include 34 countries globally.*
Data accessibility	*The data will be placed in Species360׳s Conservation Science Alliance data repository*https://www.species360.org/serving-conservation/ship-wrecks-as-reefs/

**Value of the data**

• A standardized database of worldwide intentionally deployed wrecks, from diverse information sources in a spatially explicit format including the type, material, year of deployment and estimates of the life span for each wreck.
• It can be used to analyse the potential of wrecks as artificial reefs under different conditions since each wreck׳s record has associated key Standard Level Data, such as pH, chlorophyll A concentration, calcite, and sea surface temperature from the World Ocean Database [1].
• It can provide key information to assess the role of wrecks as artificial reefs to conserve marine biodiversity, because each wreck׳s record is provided with the Euclidean distance with respect to: i) the Worlds Protected Areas [2], ii) the closest Coral Reef from the Coral Trait Database [3], and to each of the 597 aquariums member institution of the Species360 network [4].
• These data can help the prioritization of key areas for artificial reef monitoring or deployment.

## 1 Data

**Fig. 1:**
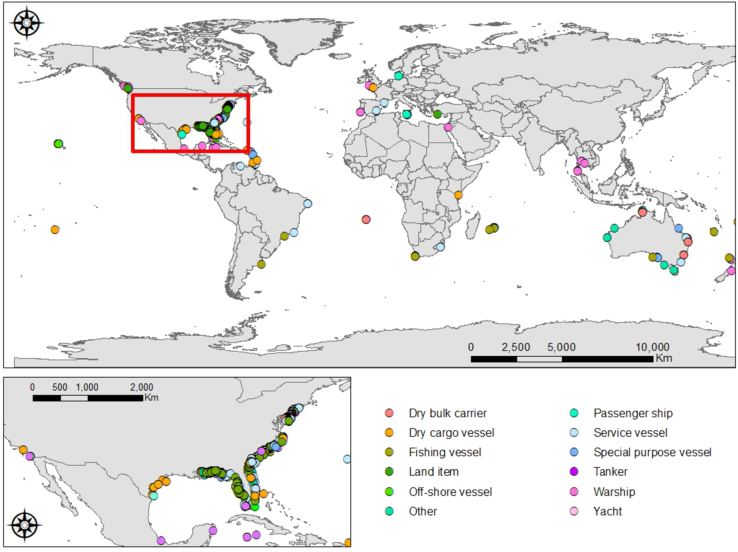
Types of intentionally deployed vessels in the dataset. Colors indicate different types of vessels.

The data have a total of 1907 records from 88 sources (Table 1 [5]). Most of them (1739 or 91%) correspond to the USA locations, while the other 9% (168) were distributed around the rest of the world (Table 2 [6]). The majority of the wrecks (1118 or 71%) were vessels (Fig. 1). For 21% (408) of the records, we do not have information on the year of deployment. Of all the deployed wrecks’ analyzed worldwide, 1739 are from the USA (Table 2 [6]).

**Table 1:** Sources of wrecks׳ location and depth. The sources include existing databases, diving guides and scientific publications.

	**Wrecks Data Sources**
1	F.D. Amaral, C.M.R. Farrapeira, S.M.A. Lira, C.A.C.Ramos. Benthic macrofauna inventory of two shipwrecks from Pernambuco Coast, Northeastern of Brazil. 2010. Revista Brasileira de Zoologia
2	Y. von Armin, O. Tyack. First observations on fish recruitment on artificial reef “Hoi Siong”. MMCS. 2003.
3	P.T. Arena, L.K.B. Jordan, R.E. Spielerl, R.E. Fish assemblages on sunken vessels and natural reefs in southeast Florida, USA. 2007. *Hydrobiologia 580*, 157–171
4	Australian Government, Department of the Environment and Energy, Australian National Shipwreck Database. 2009.http://www.environment.gov.au/heritage/historic-shipwrecks/australian-national-shipwreck-database
5	J. Brown. Artificial reefs. Document: EMD-MC-RPT-2014-0002. 2014. Environmental Management Division. Saint Helena Government.
6	D.H. Cavalcanti dos Santos, M.G.G. Silva-Cunha, M.F. Santiago, J.Z. de Oliveira Passavante. Characterization of phytoplankton biodiversity in tropical shipwrecks off the coast of Pernambuco, Brazil. 2010. *Acta bot. bras.(Online) 24(4)*: 924–934. Available from: 〈http://www.scielo.br/scielo.php?script=sci_arttext&pid=S0102-33062010000400007&lng=en&nrm=iso〉. ISSN 0102–3306. http://dx.doi.org/10.1590/S0102-33062010000400007. Accessed 20.10.2017
7	S. Chidgey, P. Crockett. The Canberra Marine Ecosystem Monitoring Program 6-Months Post Scuttling. 2010.
8	E.B. Fagundes-Netto, L.R. Gaelzer, R. Coutinho, I.R. Zalmon. Influence of a shipwreck on a nearshore-reef fish assemblages off the coast of Rio de Janeiro, Brazil. 2011. *Lat. Am. J. Aquat. Res. (Online), 39(1)*, 103–116. Available from: 〈http://www.scielo.cl/scielo.php?script=sci-arttext&pid=S0718-560X2011000100010&lng=es&nrm=iso〉. ISSN 0718-560X. http://dx.doi.org/10.4067/S0718-560X2011000100010. Accessed 20.10.2017
9	A.F. Fischer. Afundamento dos Naufrágios Mercurius, Saveiros e Taurus, caracterização e comportamento de simbiose alimentar da Ictiofauna na plataforma de Pernambuco Brasil. 2009. Universidade Federal de Pernambuco
10	A. Fukunaga, J.H. Bailey-Brock. Benthic infaunal communities around two artificial reefs in Mamala Bay, Oahu, Hawaii. 2008. *Marine Environmental Research (2007)*. doi: 10.1016/j.marenvres.2007.11.003.
11	G. Genzano, D. Giberto, C. Bremec. Benthic survey of natural and artificial reefs off Mar del Plata, Argentina, southwestern Atlantic. 2011. *Lat. Am. J. Aquat. Res., 39(3),* 553–566. doi: 10.3856/vol39-issue3-full text-15.
12	N. McDaniel. A management plan for artificial reef development in British Columbia Provincial Marine Parks. 1993. Unpublished draft report prepared for British Columbia Ministry of Environment, Lands and Parks, North Vancouver, B.C. 25p. in: B.D. Smiley. The intentional scuttling of surplus and derelict vessels: Some effects on marine biota and their habitats in British Columbia waters. 2006.
13	P.F. Morrison, Geographe Bay Artifical Reef Society Inc & Australia. Biological Monitoring of the former HMAS Swan: fifth annual report, submitted to Environment Australia. 2003. The Society, (Bunbury, W.A).
14	E. Parnell. Ecological Assessment of the HMCS Yukon Artificial Reef off San Diego, CA (USA). 2005.
15	S. Robertson. SS Taioma and SS Taupo: An analysis of fish assemblages at two artificial reefs in the Bay of Plenty, testing survey methodology and impacts of structures on sea floor sedimentology. 2012. University of Waikato. https://docs.google.com/file/d/0BxsciNHvcwTvbldDdFpacW12Z0U/edit. Accessed 20.10.2017
16	[9] G. Plunkett., Sea Dumping in Australia: Historical and Contemporary Aspects (1^st^ ed.). Canberra: Defence Publishing Service, Department of Defence.
17	M.A. Schlacher-Hoenlinger, S.J. Walker, J.W. Johnson, T.A. Schlacher, J.N.A. Hooper, M. Ekins, I.W. Banks, P.R. Sutcliffe. Biological monitoring of the ex-HMAS Brisbane artificial reef: phase II - habitat values. Technical report for the Queensland Museum. 2009.
18	M.A. Schlacher-Hoenlinger, S.J. Walker, J.W. Johnson, T.A. Schlacher, J.N.A. Hooper. Biological baseline survey of the ex-HMAS Brisbane artificial reef. Technical report for the Queensland Museum. 2006.
19	D. Stephan, D.G. Lindquist. A comparative analysis of the fish assemblages associated with old and new shipwrecks and fish aggregating devices in Onslow Bay, North Carolina. 1989. Bulletin of Marine Science, 44(2): 698–717
20	Subsea Enterprise. A biological assessment of the “*G.B. Church*” artificial reef at Princess Margaret Provincial Park. Unpublished report for the Ministry of Environment, Lands and Parks, Province of British Columbia, North Vancouver, B.C. 1994. 17p. in: B.D. Smiley. The intentional scuttling of surplus and derelict vessels: Some effects on marine biota and their habitats in British Columbia waters. 2006.
21	C. Valkenier. Unpublished survey data for the *Columbia* artificial reef, 29 August 1998, provided courtesy to Fisheries and Oceans Canada, Sidney, BC. 1998a. 1p. in: B.D. Smiley. The intentional scuttling of surplus and derelict vessels: Some effects on marine biota and their habitats in British Columbia waters. 2006.
22	C. Valkenier. Unpublished survey data for the *Saskatchewan* artificial reef, 5 September 1998, provided courtesy to Fisheries and Oceans Canada, Sidney, BC. 1998b. 1p. in: B.D. Smiley. The intentional scuttling of surplus and derelict vessels: Some effects on marine biota and their habitats in British Columbia waters. 2006.
23	C. Valkenier. Unpublished survey data for the *Mackenzie* artificial reef, 7 April 2001, provided courtesy to Fisheries and Oceans Canada, Sidney, BC. 2001. 1p. in: B.D. Smiley. The intentional scuttling of surplus and derelict vessels: Some effects on marine biota and their habitats in British Columbia waters. 2006.
24	C. Valkenier. Unpublished survey data for the *Church* artificial reef, 23 February 2002, provided courtesy to Fisheries and Oceans Canada, Sidney, BC. 2002. 1p. in: B.D. Smiley. The intentional scuttling of surplus and derelict vessels: Some effects on marine biota and their habitats in British Columbia waters. 2006.
25	S.J. Walker, T.A. Schlacher. Assessing the habitat and conservation value of a young artificial reef.
26	L. Wantiez, P. Thollot. Colonization of the F/V;Caledonie Toho 2 Wreck by a Reef-Fish Assemblage Near Noumea (New Caledonia). 2000. *Atoll Research Bulletin (485).*http://citeseerx.ist.psu.edu/viewdoc/download?doi=10.1.1.507.5400&rep=rep1&type=pdf. Accessed 20.10.2017.
27	P.H. Wendt, D.M. Knot, R.F. van Dolah. Community structure of the sessile biota on five artificial reefs in different ages. 1989. Bulletin of Marine Science, 44(3): 1106–1122
28	http://adventuredivers-spain.com/index.php/en/dive-sites/wreck-dives. Accessed 10.04.2016.
29	http://artificialreefsocietybc.ca/annapolis.html. Accessed 05.09.2016.
30	http://artificialreefsocietybc.ca/boeing-737.html. Accessed 05.09.2016.
31	http://artificialreefsocietybc.ca/cape-breton.html. Accessed 05.09.2016.
32	http://artificialreefsocietybc.ca/chaudiere.html. Accessed 05.09.2016.
33	http://artificialreefsocietybc.ca/columbia.html. Accessed 05.09.2016.
34	http://artificialreefsocietybc.ca/g-b-church.html. Accessed 05.09.2016.
35	http://artificialreefsocietybc.ca/mackenzie.html. Accessed 05.09.2016.
36	http://artificialreefsocietybc.ca/saskatchewan.html. Accessed 05.09.2016.
37	http://british-virgin-islands.greatestdivesites.com/cooper_island/mv_inganess_bay. Accessed 06.09.2016.
38	http://coastalgadnr.org/sites/uploads/crd/images/Reef/GeorgiaOffshoreReefWeb.pdf. Accessed 10.09.2016.
39	http://diveadvisor.com/jersey/mauve-(la)-ss. Accessed 10.09.2016.
40	http://diveadvisor.com/sub2o/now-thats-a-huge-aircraft-diving-the-dakota-dc3-in-southern-turkey. Accessed 10.09.2016.
41	http://dlnr.hawaii.gov/dar/files/2014/04/ARCoords.pdf. Accessed 12.09.2016.
42	http://myfwc.com/media/131585/reefs.pdf. Accessed 12.09.2016.
43	http://oceantribe.co/mv-dania-east-africas-number-1-wreck-dive-site/. Accessed 12.09.2016.
44	http://portal.ncdenr.org/c/document-library/get-file?uuid=24160156-4b96-49e6-9126-4fa488b49cbb&groupId=38337. Accessed 15.09.2016.
45	http://portal.ncdenr.org/c/document-library/get-file?uuid=b70dfece-9e83-40fd-9a33-dcf6d1a13990&groupId=38337. Accessed 15.09.2016.
46	http://scubaclubcozumel.com/index.php/felipe-xicotencatl. Accessed 10.09.2016.
47	http://thailandliveaboards.com/thailand-dive-sites/krabi-diving/. Accessed 10.09.2016.
48	http://thedecostop.com/wrecks/db/form.php?table_name=wrecks. Accessed 10.04.2016.
49	http://www.adiscuba.com/dive-sites/199/south+africa/south+africa/durban/. Accessed 10.04.2016.
50	http://www.aquaexplorers.com/artificialreefprogramny.htm. Accessed 13.09.2016.
51	http://www.aquaexplorers.com/gpsnynjartificialreef.pdf. Accessed 13.09.2016.
52	http://www.capescuba.co.za/dive-the-mv-aster-with-cape-scuba-club/. Accessed 10.04.2016.
53	http://www.cawreckdivers.org/wrecks/yukon.htm. Accessed 10.04.2016.
54	http://www.cita.ky/capt-keith-tibbetts. Accessed 10.09.2016.
55	http://www.dive365cayman.com/doc-poulson-wreck. Accessed 10.09.2016.
56	http://www.dive365cayman.com/kittiwake-cayman. Accessed 10.09.2016.
57	http://www.dive365cayman.com/oro-verde. Accessed 10.09.2016.
58	http://www.diveboard.com/explore/spots/reunion/saint-leu-L54ihjW/antonio-lorenzo-S2SNhfl. Accessed 10.09.2016.
59	http://www.diveboard.com/explore/spots/russian-federation/central-LM8puM/gran-roque-SuESLz. Accessed 10.09.2016.
60	http://www.divebuddy.com/divesite.aspx?DiveSiteID=5157. Accessed 15.09.2016.
61	http://www.divebuddy.com/divesite/3766/franjack-guadeloupe/. Accessed 15.09.2016.
62	http://www.divebuddy.com/divesite/3900/theos-wreck-bahamas. Accessed 15.09.2016.
63	http://www.divebuddy.com/divesite/5501/mv-hildur-grenada/. Accessed 15.09.2016.
64	http://www.divesitedirectory.co.uk/dive_site_the_cook_islands_rarotonga_wreck_mataora.html. Accessed 10.09.2016.
65	http://www.divesitedirectory.co.uk/dive_site_uk_england_southwest_wreck_hms_scylla.html. Accessed 10.09.2016.
66	http://www.dmr.state.ms.us/index.php/marine-fisheries/artificial-reef/74-offshore-reefs. Accessed 17.09.2016.
67	http://www.dnr.sc.gov/artificialreefs/docs/ReefGuide2015.pdf. Accessed 17.09.2016.
68	http://www.dnrec.delaware.gov/fw/Fisheries/Documents/2015-16%20DELAWARE%20REEF%20GUIDE.pdf. Accessed 17.09.2016.
69	http://www.dyk-sydfyn.dk/2-dive-spot-m-f-%C3%A6r%C3%B8sund.html?mcat=-1&caction=showspot&itemid=22. Accessed 17.09.2016.
70	http://www.famouspublishing.co.za/ridge/wreck-dive-off-umhlanga/, Accessed 17.09.2016.
71	http://www.mmcs-ngo.org/en/projects/artificial-reefs.aspx. Accessed 08.09.2016.
72	http://www.mytobago.info/diving01b.php. Accessed 08.09.2016.
73	http://www.navsource.org/archives/10/14/14469.htm. Accessed 08.09.2016.
74	http://www.navsource.org/archives/11/02255.htm. Accessed 08.09.2016.
75	http://www.nj.gov/dep/fgw/pdf/reefs/reef_guide.pdf. Accessed 10.09.2016.
76	http://www.oceanrevival.org/en/projecto/local-mergulho.html. Accessed 10.09.2016.
77	http://www.outdooralabama.com/artificial-reefs. Accessed 10.09.2016.
78	http://www.pattayadivers.com/2012/10/26/htms-mataphon/. Accessed 11.09.2016.
79	http://www.pdsa.org.mt/index.php/component/content/article/11-diving-locations-content/72-mv-imperial-eagle. Accessed 11.09.2016.
80	http://www.pdsa.org.mt/index.php/component/content/article/11-diving-locations-content/75-tugboat-p31. Accessed 11.09.2016.
81	http://www.pdsa.org.mt/index.php/component/content/article/11-diving-locations-content/77-mv-xlendi. Accessed 11.09.2016.
82	http://www.pdsa.org.mt/index.php/component/content/article/11-diving-locations-content/78-karwela. Accessed 11.09.2016.
83	http://www.pdsa.org.mt/index.php/component/content/article/11-diving-locations-content/79-mv-cominoland. Accessed 11.09.2016.
84	http://www.scubadivingmalta.co.uk/assetts/Maltainserttugboat2.pdf. Accessed 12.09.2016.
85	http://www.scubastevesdiving.com/dive-sites#!. Accessed 12.09.2016.
86	http://www.scubatravel.co.uk/americas/diving-brazil.html. Accessed 12.09.2016.
87	http://www.smsepub.com/article/Dive%3A_Wreck_Diving/801592/77284/article.html. Accessed 10.09.2016.
88	http://www.statiapark.org/parks/marine/img/statia_dive_map.pdf. Accessed 11.09.2016.
89	http://www.tablebaydiving.com/dive-site-sas-pietermaritzburg.shtml. Accessed 11.09.2016.
90	http://www.tablebaydiving.com/dive-site-smitswinkel-bay-wrecks.shtml. Accessed 17.09.2016.
91	http://www.thaiwreckdiver.com/htms-chang-lst-2-uss-lincoln-county-lst-898.htm. Accessed 17.09.2016.
92	http://www.touria.nl/bestanden/malta/Wrakken_Malta.pdf?1824951652. Accessed 11.09.2016.
93	http://www.wannadive.net/spot/Central_America/Guadeloupe/L_Augustin_Fresnel_II/index.html. Accessed 11.09.2016.
94	https://en.wikipedia.org/wiki/HMNZS_Canterbury_(F421). Accessed 13.09.2016.
95	https://en.wikipedia.org/wiki/HMNZS_Tui_(1970). Accessed 13.09.2016.
96	https://en.wikipedia.org/wiki/HMNZS_Waikato_(F55). Accessed 13.09.2016.
97	https://en.wikipedia.org/wiki/HMNZS_Wellington_(F69). Accessed 13.09.2016.
98	https://en.wikipedia.org/wiki/Maltese_patrol_boat_P29. Accessed 13.09.2016.
99	https://en.wikipedia.org/wiki/MV_Rozi. Accessed 13.09.2016.
100	https://en.wikipedia.org/wiki/Rainbow_Warrior_(1955). Accessed 13.09.2016.
101	https://en.wikipedia.org/wiki/Um_El_Faroud. Accessed 13.09.2016.
102	https://nrm.dfg.ca.gov/FileHandler.ashx?DocumentID=30217&inline=true. Accessed 10.09.2016.
103	https://tpwd.texas.gov/publications/pwdpubs/media/pwd_br_v3400_0424.pdf. Accessed 10.09.2016.
104	https://tpwd.texas.gov/publications/pwdpubs/media/pwd_rp_v3400_0491.pdf. Accessed 10.09.2016.
105	https://www.doi.gov/sites/doi.gov/files/migrated/deepwaterhorizon/adminrecord/upload/ENVIRONMENTAL-REMEDIATION-OF-THE-USTS-TEXAS-CLIPPER-FOR-USE-AS-AN-ARTIFICIAL-REEF-IN-THE-GULF-OF-MEXICO-prepared-by-Texas-Parks-and-Wildlife-Dept-Sept-7–2007-2.pdf. Accessed 11.09.2016.
106	https://www.scubacrowd.com/dive-site/palamos/pecio-boreas/107. Accessed 10.03.2016.

**Table 2:** Overview of the distribution of intentionally deployed wrecks in the USA and rest of the world.

**Location**	**Total**	**Dry bulk carrier**	**Dry cargo vessel**	**Fishing vessel**	**Land item**	**Off-shore vessel**	**Other**	**Passenger ship**	**Service vessel**	**Special purpose vessel**	**Tanker**	**Warship**	**Yacht**
USA	1739	12	126	71	548	2	118	8	604	23	39	183	5
Rest of the world	168	18	9	16	3	0	27	8	45	13	1	27	1
Total	1907	30	135	87	551	2	145	16	649	36	40	210	6

**Table 3:** Number of deployed wrecks by country.

**Location**	**Total**	**Dry bulk carrier**	**Dry cargo vessel**	**Fishing vessel**	**Land item**	**Other**	**Passenger ship**	**Service vessel**	**Special purpose vessel**	**Tanker**	**Warship**	**Yacht**
**Argentina**	**1**	0	0	1	0	0	0	0	0	0	0	0
**Australia**	**64**	16	0	2	0	14	2	21	7	0	1	1
**Barbados**	**1**	0	1	0	0	0	0	0	0	0	0	0
**Bermuda Island**	**1**	0	0	0	0	0	0	1	0	0	0	0
**Brazil**	**7**	0	0	1	0	0	0	6	0	0	0	0
**British Virgin Islands**	**2**	0	1	0	0	0	0	1	0	0	0	0
**Canada**	**8**	0	1	0	1	0	0	1	0	0	5	0
**Cayman Islands**	**4**	0	0	0	0	0	0	0	1	0	3	0
**Cook Islands**	**1**	0	1	0	0	0	0	0	0	0	0	0
**Denmark**	**1**	0	0	0	0	0	1	0	0	0	0	0
**Fiji**	**1**	0	1	0	0	0	0	0	0	0	0	0
**Great Britain**	**2**	0	1	0	0	0	0	0	0	0	1	0
**Grenada**	**1**	0	1	0	0	0	0	0	0	0	0	0
**Guadeloupe**	**1**	0	0	0	0	0	0	0	1	0	0	0
**Israel**	**1**	0	0	0	0	0	0	0	0	0	1	0
**Kenya**	**1**	0	1	0	0	0	0	0	0	0	0	0
**Malta**	**11**	0	0	0	0	0	4	6	0	1	0	0
**Mauritius**	**13**	0	0	1	0	12	0	0	0	0	0	0
**Mexico**	**2**	0	0	0	0	0	0	0	0	0	2	0
**New Caledonia**	**1**	0	0	1	0	0	0	0	0	0	0	0
**New Zealand**	**6**	0	0	1	0	0	0	2	0	0	3	0
**Portugal**	**4**	0	0	0	0	0	0	2	0	0	2	0
**Reunion**	**1**	0	0	1	0	0	0	0	0	0	0	0
**Saint Helena**	**6**	2	0	2	1	1	0	0	0	0	0	0
**Saint Lucia**	**2**	0	0	0	0	0	0	0	2	0	0	0
**South Africa**	**10**	0	0	5	0	0	0	1	1	0	3	0
**Spain**	**3**	0	0	0	0	0	0	2	0	0	1	0
**St. Eustasius Island**	**2**	0	0	1	0	0	0	0	1	0	0	0
**Thailand**	**5**	0	0	0	0	0	0	0	0	0	5	0
**The Bahamas**	**1**	0	1	0	0	0	0	0	0	0	0	0
**Trinidad and Tobago**	**2**	0	0	0	0	0	1	1	0	0	0	0
**Turkey**	**1**	0	0	0	1	0	0	0	0	0	0	0
**Venezuela**	**1**	0	0	0	0	0	0	1	0	0	0	0

### 1.1 Distribution of wrecks in the USA

**Fig. 2:**
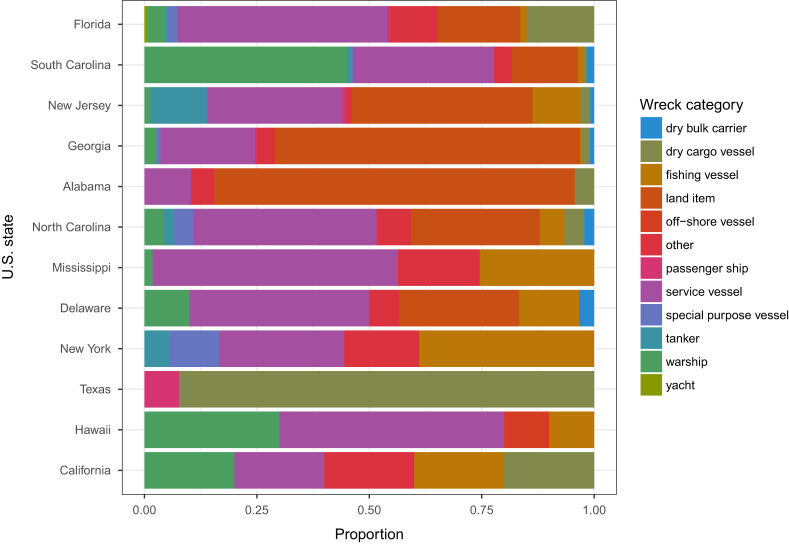
Types of intentionally deployed wrecks in 12 of the American states.

**Fig. 3:**
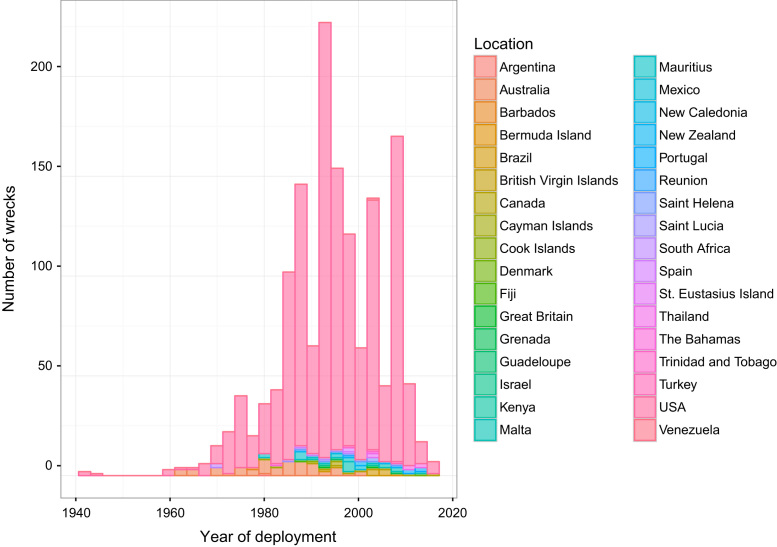
Number of intentionally deployed wrecks by location and year.

More than half of the wreck׳s records (68%) in the USA are vessels. Subway cars/boxcars, automobile bodies, battle tanks, aircrafts, and submarines constitute the remaining 548 wrecks in the data. Florida has the highest number of wreck records (43%). The dominant type of wreck data in all states, except Georgia and Alabama, was a vessel (Fig. 2 [5]). In Texas, North Carolina, California, Virginia and South Carolina more wrecks were sunk in the period before 1990 (Fig. 3 [6]).

### 1.2 Distribution of wrecks in the rest of the world

**Fig. 4:**
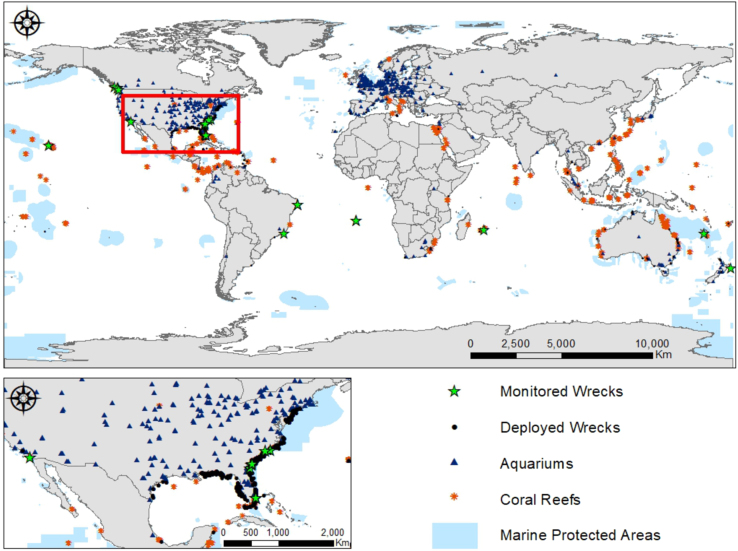
Global distribution of monitored and deployed wrecks, aquariums, coral reefs and marine protected areas.

**Fig. 5:**
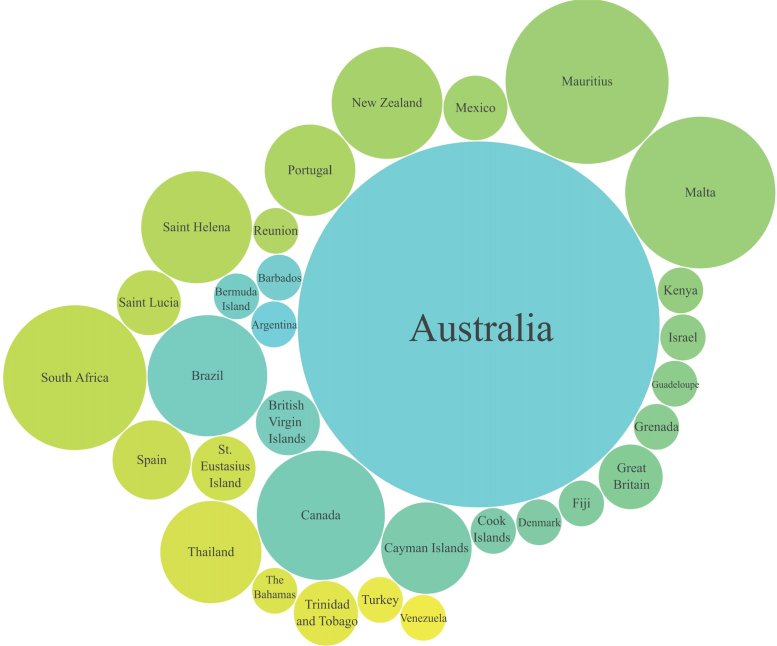
Comparison of the number of intentionally deployed wrecks globally, excluding the USA. The size of the circles is proportional to the number of sunken wrecks, with a minimum of 1 and maximum of 64.

The data contain 168 wreck records distributed in 32 countries around the world (Tables S1 and 3, Fig. 4 [7]). Most of the deployed vessels (38% of the 168) are in Australia (Fig. 5 [2]).

## 2 Experimental Design, Materials and Methods

We compiled data of 1907 intentionally deployed wrecks to serve as artificial reefs from diverse sources, including local artificial reef databases, scientific publications, and diving web sites (Table 1). Publications include scientific articles, monitoring reports, student theses and dissertations. The initial search for websites or publications with wrecks included mainly the use of the *Google* search engine, including *Google Scholar* and *PubMed*, with one or a combination of the following key words and expressions: shipwreck, artificial reef, sunken vessels, intentionally deployed. Later, we specified the search by adding a location, e.g., artificial reefs Europe, intentionally deployed vessels USA, shipwrecks as artificial reefs Australia, etc. Regarding wrecks located in the USA, the search was further specified using the key expression artificial reef with an addition of the particular state, e.g. artificial reefs Florida, artificial reefs Georgia, etc. In the majority of cases, the result of this search led to an official web page of the artificial reef program in the state in question. Furthermore, these web sites contained lists of all artificial reefs in the state. From these lists, we extracted information regarding only vessels and other types of wrecks. Regarding wrecks as artificial reefs in Australia, we used two main sources of information: 1) the Australian National Shipwreck Database [8], and 2) a report regarding sea dumping in Australia, prepared for the Australian Government, Department of Environment and Heritage [9]. We obtained data regarding wrecks as artificial reefs in the rest of the world from diving websites and scientific publications. We also gathered information about 1901 sites of wrecks of various types intentionally deployed globally as artificial reefs. The main fields included in the database were: name of the wreck and/or the reef site, year of deployment, type of wreck, location, coordinates, depth, accuracy of the coordinates (when provided), last update of the information, source of the information and notes (Table 4). The coordinates were given in decimal degrees to allow the direct use of these data with other spatial information.

**Table 4:** Description of the variables in the main dataset.

ID	Description
Name	Name of the wreck
Year.of.deployment	Year of deployment
Type.of.wreck	High detail level of wreck category
Location.Country	Name of the country
Location.City.State.Province	Name of the City
Location.Water.Body	Name of the water bodies
Depth	Recorded depth of the wreck, in meters
Latitude	Latitude ISO 6709, in decimal degrees
Longitude	Longitude ISO 6709, in decimal degrees
Accuracy	Level of position accuracy
Last.update	Last wreck information update
Source	Internet reference link
Notes	Complementary information on the wreck location
ISO.Country.Code	ISO 3166, 3 characters code of country or territory
ISO.Country.Code.1	ISO Numeric Code UN M49 Numerical Code
ISO.Country.Code.state.province.	ISO 3166, 3 characters code of country and province
ID	Wreck ID
Wreck.Category	General category of the wreck
Protected.Area	Boolean value, 0 = not in a protected area, 1 = in a protected area
Name.Protected.Area	Name of the protected area
Near.Coral.ID	Identification of the nearest coral reef according to [3].
Distance.Coral	Distance, in km, to the nearest coral reef.
Near.Aquarium.Name	Institution name of the nearest aquarium part of Species360 [4].
Distance.Aquarium	Distance, in km, to the nearest aquarium.
Last.update.Year	Year of the last update
Last.update.Month	Month of the last update
Last.update.Day	Day of the last update
Purpose.of.deployment	Published purpose of the deployment
Method.of.sinking	Method of wreck sinking
Baseline.survey	Boolean value
Monitoring	Monitored parameters details
Method.of.monitoring	Monitored parameters method
Research.outcome	Result from monitoring
Life.span.years	Estimated lifespan of the wreck
Source.1	Reference of the monitoring
Min.Est.Lifespan	Minimum estimated wreck lifespan
Max.Est.Lifespan	Maximum estimated wreck lifespan

The data contain records of 130 types of wrecks, as described in the source, which we aggregated into 12 categories (Table 5). Each wreck record contains the citation, the latitude and longitude, the type of structure and the lifespan of the wreck to serve as an artificial reef (Table S1 & S2). We calculated the wrecks lifespan based on the type of structure and material by using the estimates provided by 36 studies that monitor the colonization of the artificial reefs (see Table 1 for the source of those studies). For these 36 wrecks we included the following information: estimates of lifespan as artificial reefs, purpose of deployment, method of sinking, baseline study before monitoring, the purpose of monitoring, the method of monitoring, and a brief summary of monitoring outcomes.

**Table 5:** Aggregated wreck categories, based on Knud E. Hansen^a^.

**Category**	**Example of artefact**
passenger ship	ferry, passenger/cargo ship
dry cargo vessel	cargo ship, freighter, liberty ship
tanker	oil tanker, fuel barge, oil field supply vessel
dry bulk carrier	iron lighter, steel barge, wooden lighter
special purpose vessel	cable ship, buoy tender, cable layer vessel
service vessel	hydrographic ship, push boat, fireboat
fishing vessel	fishing trawler, clam boat, shrimp boat
off-shore vessel	submarine
yacht	yacht
land item	army tank, bus, aircraft, railroad boxcar
warship	destroyer, aircraft carrier, armoured personnel carrier
other	drydock, sailboat, powerboat, tour boat

a Knud E. Hansen: http://www.knudehansen.com/key-services/general-naval-architecture/vessel-types/

**Fig. 6:**
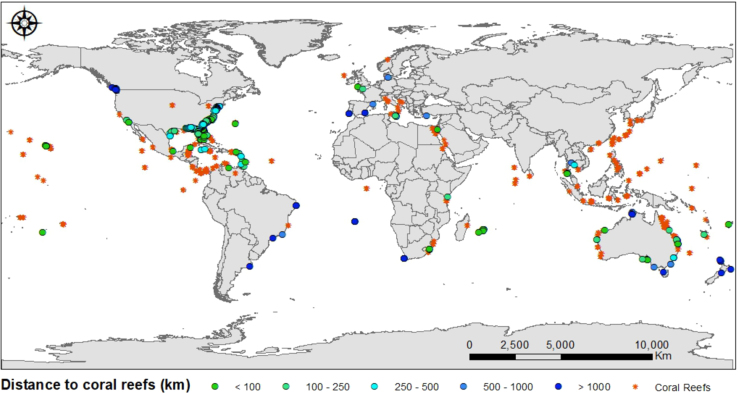
Global distribution of coral reefs (orange dots), as reported in Madin et al. [3] and its distance from deployed wrecks in kilometers. Each blue and green colored dot represents a range of distance.
ii) Indicate if the wreck is located within a protected area, from the World Database on Protected Areas [2] (Table S1).
iii) Estimate the distance of wrecks to aquariums and zoological institutions members of Species360. The Species360 global network is a non-governmental organization that manages the Zoological Information Management System (ZIMS) [4]. ZIMS is a real-time database with standardized and shared information of 21000 species from more than 1100 zoos and aquariums institutions, of which 54% report having aquatic species (Fig. 7).

We extracted biophysical marine factors in each wreck location from Feldman & McClain [10] and from the World Ocean Database [1] (Table 6). This information includes key Standard Level Data, such as pH, chlorophyll A concentration, calcite, and sea surface temperature. We used ArcGIS version 10.5.1. [11] to:

i) Calculate the Euclidean distance between coral reefs and deployed wrecks by using the data on corals’ location from the Coral Traits Database [3], which contain species-specific biogeographic locations (Fig. 6).

**Fig. 7:**
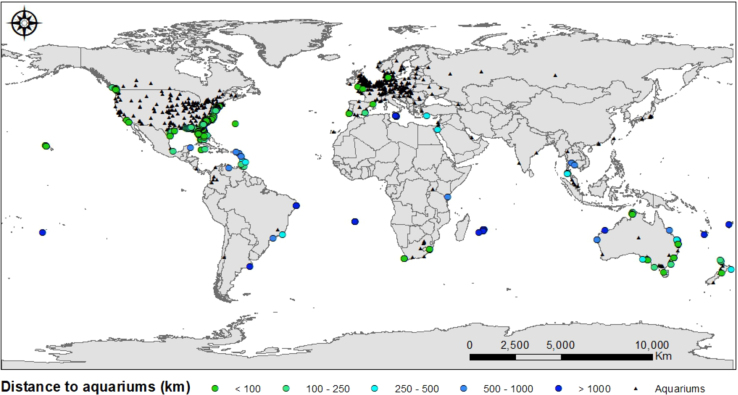
Global distribution of aquarium members of the Species360 network (black dots) and their distance from deployed wrecks in kilometers. Each blue and green colored dot represents a range of distance.

**Table 6:** Detailed description of the biophysical marine factor variables on the location of wrecks from Feldman & MacClain [10] and from Levitus [1].

ID	Variable name (unit)	Original Spatial Resolution	Sensor	Data	Temporal range	Brief description
calcite	Calcite concentration (mol/m³)	5 arcmin (9.2 km)	Aqua-MODIS	Seasonal climatologies	2002 - 2009	Calcite concentration indicates the concentration of calcite (CaCO3) in oceans
chlomax	Chlorophyll A concentration (mg/m³)	5 arcmin (9.2 km)	Aqua-MODIS	Monthly climatologies	2002–2009	Chlorophyll A concentration indicates the concentration of photosynthetic pigment chlorophyll A (the most common “green” chlorophyll) in oceans. Please note that in shallow water these values may reflect any kind of autotrophic biomass.
chlomean						Mean value of chlorophyll
chlomin						Minimum value of chlorophyll
chlorange						Range of values of chlorophyll
cloudmax	Cloud fraction (%)	6 arcmin (11 km)	Terra-MODIS	Monthly images	2005–2010	Maximum cloud fraction. It indicates how much of the earth is covered by clouds.
cloudmean						Mean cloud fraction
cloudmin						Minimum cloud fraction
damax	Diffuse attenuation coefficient at 490 nm (m-1)	5 arcmin (9.2 km)	Aqua-MODIS	Monthly climatologies	2002–2009	The diffuse attenuation coefficient is an indicator of water clarity. It expresses how deeply visible light in the blue to the green region of the spectrum penetrates in to the water column.
damean						Mean diffuse attenuation coefficient
damin						Minimum diffuse attenuation coefficient
parmax	Photosynthetically Available Radiation (Einstein/m²/day)	5 arcmin (9.2 km)	SeaWiFS	Monthly climatologies	1997–2009	Photosynthetically Available Radiation (PAR) indicates the quantum energy flux from the Sun (in the spectral range 400–700 nm) reaching the ocean surface.
parmean						Mean Photosynthetically Available Radiation (PAR)
sstmax	Sea Surface Temperature (°C)	5 arcmin (9.2 km)	Aqua-MODIS	Monthly climatologies	2002–2009	Sea surface temperature is the temperature of the water at the ocean surface. This parameter indicates the temperature of the topmost meter of the ocean water column.
sstmean						Mean Sea surface temperature
sstmin						Minimum Sea surface temperature
sstrange						Range Sea surface temperature

The data presented here do not cover all existing, deliberately sunken wrecks; the selection is based mainly on the availability of data either online or in publications in the English language. The quality and accuracy of the presented data entirely depend on the accurate, up-to-date information contained in the source documents. For quality control, we checked all data entries by at least one person who was not the main responsible for data input. For that, two people had access to the dataset at the same time: one person attributed a random record number to the other one, who was responsible to find the relevant data from the source person (such as: coordinates, name, year of deployment, depth). The two persons cross-referenced the data with the source to check for errors. To provide visualization of the data in the presented database we generated maps using ArcGIS [11].
